# Simulation of defects, flexibility and rupture in biopolymer networks

**DOI:** 10.1039/d1ra07262e

**Published:** 2022-01-13

**Authors:** Matthew H. J. Bailey, Mark Wilson

**Affiliations:** Department of Chemistry, Physical and Theoretical Chemistry Laboratory, University of Oxford South Parks Road Oxford OX1 3QZ UK mark.wilson@chem.ox.ac.uk

## Abstract

Networks of biopolymers occur often in nature, and are vulnerable to damage over time. In this work, a coarse grained model of collagen IV molecules is applied in a 2D hexagonal network and the mechanisms by which these networks can rupture are explored. The networks are stretched linearly in order to study their structural limits and mechanism of rupture over timescale of up to 100 μs. Metrics are developed to track the damage networks suffer over time, and qualitatively analyse ruptures that occur. Further simulations repeatedly stretch the networks sinusoidally to mimic the *in vivo* strains. Defects of increasing levels of complexity are introduced into an ordered network, and their effect on the rupturing behaviour of the biopolymer networks studied. The effect of introducing holes of varying size in the network, as well as strips of finite width to mimic surgical damage are studied. These demonstrate the importance of the flexibility of the networks to preventing damage.

## Abstract 2

We use a coarse grained polymer model and a simple graph representation to introduce defects into a biopolymer network, then cause them to rupture.

## Graphical abstract

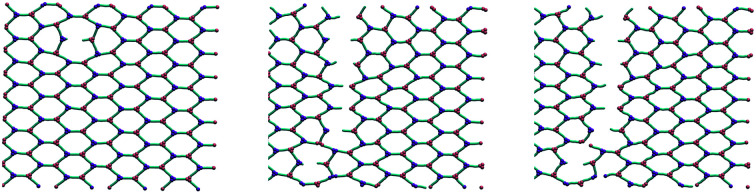

## 1 Introduction

Networks of biopolymers are present in numerous forms across the human body and there has been much scientific interest in trying to understand their properties and, in particular, how they react to being damaged. One such important biopolymer network that has been studied is collagen IV in the ocular lens capsule.^1,2^ The balance between order and disorder in the lens capsule leads to a range of complex mechanical properties which may have significant consequences for human visual function, and which have been a topic of research from different perspectives for over a century.^3^ Furthermore, the structure of collagen biopolymer networks changes over time, leading to the lens capsule stiffening and consequentially causing a loss of ability to accommodate different focal depths.^4,5^

The intra-ocular lens in humans is sometimes surgically replaced, in cataract surgery or following blunt trauma.^1,6^ The replacement artificial lens can sometimes be designed to allow focal accommodation where the natural lens had lost that ability.^7,8^ This surgery necessitates a small circular hole being cut in the collagen of the lens capsule. Sadly in approximately 4.00% of surgeries the lens capsule ruptures, leading to a serious loss of visual acuity for the patient.^9,10^ The rupture of the lens capsule arises from surgical damage to the biopolymer network—damage to collagen networks in general has been studied using crosslinked semiflexible biopolymer networks,^11,12^ and the rupture of the lens capsule has been studied using finite element methods and measured experimentally.^6,13^ As yet no link has been made between the micro-scale behaviour of collagen networks and the macro-scale rupture of the lens capsule, in part as it is extremely difficult to capture images of the dynamical rupturing process as it occurs over time scales of approximately 1.00 ms.^6^

This work aims to provide a mechanistic study of the rupture of collagen networks using a combination of a simplified graph representation and a coarse grained polymer model. The simplified graph representation allows for a robust exploration of how structural features are linked to mechanisms of rupture, and the coarse grained polymer model allows access to relatively long simulation timescales.^12^ A graph based model also provides a powerful way to include well-defined defects, and combine individual defects systematically. The coarse grained polymer model combined with the graph model means that we can observe the dynamics and mechanical response of biopolymer networks containing a range of defects.

## 2 Computational methods

### 2.1 Defect introduction

**Fig. 1:**
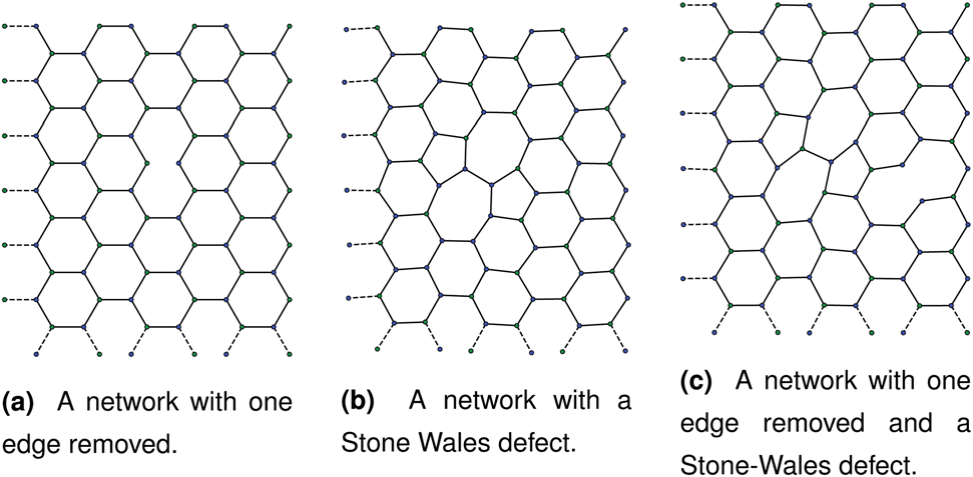
Three networks with different combinations of defects. Each panel corresponds to dimensions of approximately 1.80 μm by 2.40 μm.

Our initial hypothesis was that rupture propagated from a weak site within a biopolymer network, which would be found close to an existing defect. Before testing the mechanism of rupture, we therefore require a method of reproducibly introducing realistic defects into a network structure. We start with a 2D hexagonal network where each node has a coordination number *k* = 3, assuming a simplified model with no crossings between molecules for computational simplicity. 2D networks with appropriate connectivities have been effectively used to scan wide parameter spaces with lower computational cost than 3D networks, while capturing the relevant physics—for example, 2D triangular networks have been used by Jansen and coworkers.^14^ The average connectivity of the collagen network has been shown to be a significant factor in the rupturing properties.^15^ A 2D hexagonal network of collagen molecules has been used successfully by Burd to mimic mechanical properties of the eye, and is based on experimental observations from Barnard *et al.* and Yurchenco and Ruben.^16–18^ The ordered network represents the structure of collagen that has formed on an ordered scaffold in the eye,^19^ and is more interpretable mechanistically than random network models such as the Mikado model.^20^ Starting with a hexagonal network we introduce defect in two ways. The first way to introduce a defect is to remove edges one at a time in a graph representation of the hexagonal network. Edges to be deleted were chosen at random from the hexagonal network. After the randomly selected edges were removed, the network was scanned to check for dangling nodes with coordination number *k* = 1. Any edges leading to dangling nodes and nodes involved in zero edges were removed recursively until no more *k* = 1 nodes remained. A single bond removal is shown in Fig. 1a.

**Fig. 2:**
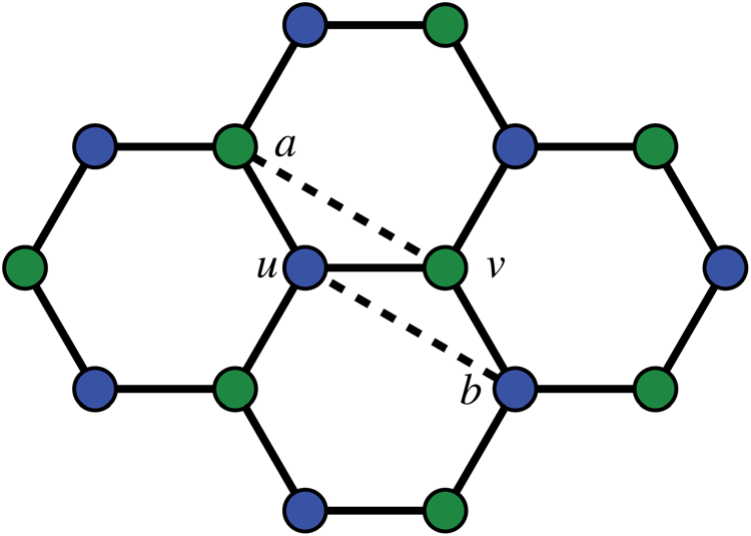
A schematic bond switching step; the edges (*a*, *u*) and (*v*, *b*) are deleted and the new dashed edges (*a*, *v*) and (*u*, *b*) are added. Each edge is an abstract representation of a single collagen IV molecule, which has a length of about 300 nm. The colours further highlight the defect with two blue-green pairs being replaced by blue-blue and green-green pairs. This transforms four hexagons into two heptagons and two pentagons. Geometry optimisation then relaxes the nodes into less strained positions, as can be seen in Fig. 1b.

The second way to introduce a defect is using an extended Wooten–Winer–Weaire bond switching algorithm as extended by Ormrod Morley and Wilson and shown to be useful to generate high-quality continuous random networks by Bailey *et al.*^21–23^ The bond switching step is shown schematically in Fig. 2 and performed by the following algorithm:

• Pick a random edge (*u*, *v*).

• Pick two new edges (*a*, *u*) and (*v*, *b*), ensuring that *a*, *b*, *u*, *v* are all unique. Ensure that (*a*, *u*) and (*v*, *b*) are in different polygons.

• Delete edge (*a*, *u*) and (*v*, *b*) and add edges (*a*, *v*) and (*u*, *b*).

• Locally optimise the geometry.

The geometry was locally optimised using scipy, applying a harmonic potential to the bonds and no angular potential in the interests of computational speed.^24^ A single bond switching move introduces a defect known as a Stone–Wales defect into a hexagonal network, and is shown schematically in Fig. 1b.^25^ Stone–Wales defects have been studied in great detail as a major type of defect in 2D silica glasses.^26^

These categories of defects can be combined, performing multiple bond switching moves and edge removals to create more complex defect structures, for example the combined bond switch and edge removal shown in Fig. 1c. Furthermore, the combined defects can be correlated or uncorrelated with one another to lead to more complex scenarios; in this work we choose uncorrelated defects unless otherwise stated. However, the introduction of multiple defects in the graph representation required optimisation to a local energy minimum. At nodes, the energy of an edge was given by a simple harmonic potential

1 *U* = *k*_bond_(*r* − *r*_eqm_)^2^,

with equilibrium length *r*_eqm_ = 1.0 and the angular energy between neighbours around one node given by a cosine potential

2 *U* = *K*_angle_(cos(*θ*) − cos(*θ*_eqm_))^2^

where 
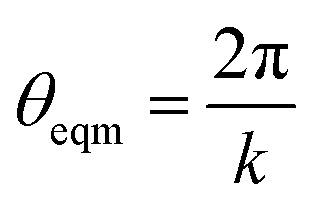
 with *k* being the coordination number (or degree) of the central node. This was chosen because it mimics the DREIDING potential often used for polymer simulation.^27^

The minimisation was performed using scipy's BFGS routines,^24^ and was accelerated by including information on the Jacobian; the Jacobian is intuitive from a chemical perspective as the negative of the vector forces. The bond Jacobian elements are given by3
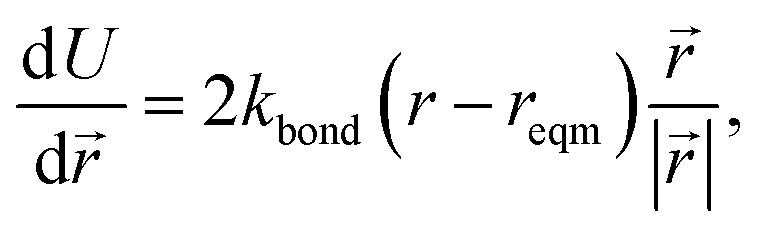
and the angular Jacobian elements are given by4
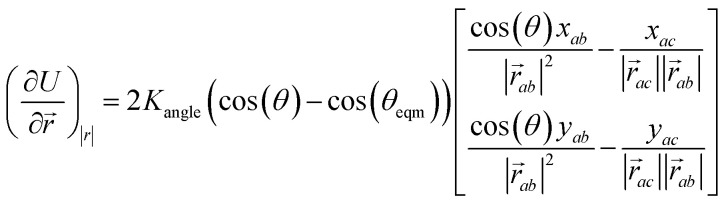
where 
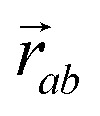
 is the vector from the central node *a* to neighbouring node *b* that has components *x*_*ab*_ and *y*_*ab*_; the vector 
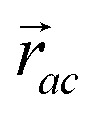
 has similar meaning for the *a* to *c* edge. The defect-containing graphs were converted into polymers, and the polymer arrangements energetically minimised using LAMMPS's implementation of the Polak–Ribiere Conjugate Gradient method.^28^ The bond switched networks are much more coarse grained than a polymer network. This makes converting a graph based network that is the output of the bond switching procedure into coarse grained entities relatively hard, as new information must be generated *via* a heuristic. The heuristics we have developed work well for idealised network structures with regular polygons, but work less well for irregular polygons.

The conversion algorithm follows these steps:
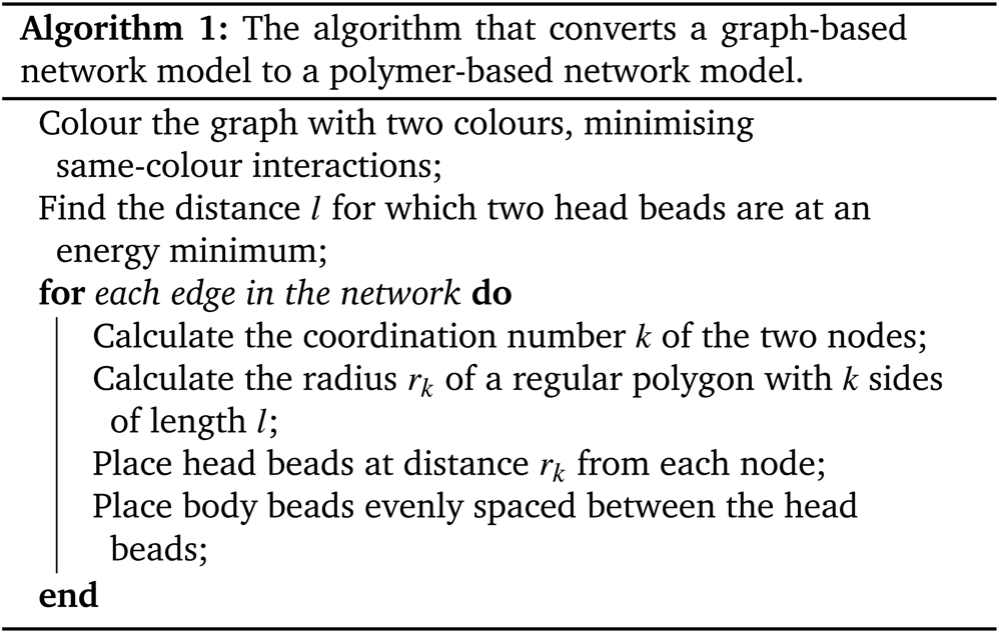


### 2.2 Polymer model

Tropocollagen molecules in a network can suffer two major modes of damage: damage at cross-linking sites (represented as clusters of head groups in this model) or damage within a molecule.^29^ The damage at cross-linking sites is accounted for by the presence of Lennard–Jones pairwise interactions between head groups, which allows for damage to be repaired. The potential model for polymer interactions has been described in previous work by Bailey and Wilson.^12^ The core components are coarse grained polymers made up of six beads, where the two ends are “head beads” and the remaining beads are “body beads”.

• Body beads repel other body beads using a truncated Lennard–Jones interaction.

• Head beads attract other head beads using a Lennard–Jones interaction.

• Adjacent pairs of beads in a polymer are held together with a bonding potential (here a shifted Morse potential).

• Adjacent triplets of beads in a polymer are held straight by an angular potential (here a cosine squared potential).

The polymers are simulated using LAMMPS in a 2D plane and a Langevin thermostat. The specific parameters used in this model are varied to explore the total landscape of rupturing behaviour and discussed in more detail in each section. The exploration of a range of parameters allows the study of damage to hexagonal biopolymer networks (like collagen IV) under different conditions, where experimental data to fit to may be sparse. Where not otherwise specified, the simulations use an attractive Lennard–Jones well depth of *ε*_HH_ = 16.5 × 10^−21^ J (1200 K *k*_B_),a repulsive Lennard–Jones energy parameter of *ε*_BB_ = 64.0 × 10^−21^ J and an angular energy parameter of *K*_*θ*_ = 200 × 10^−21^ J. The Langevin thermostat is set in the range 100 K to 300 K with a damping parameter of 1.00 ns. However, rupture of a covalent bond in a single tropocollagen molecule cannot be repaired and must be modelled differently. To account for the effect of bonds breaking, we use fix bond/break feature of LAMMPS. This checks all bonds every *N* steps to check if any bond lengths are greater than a cutoff distance *r*_c_. If a bond is longer than *r*_c_, it is removed and the neighbour lists representing bonds and angular bonds are recalculated. In this work, we choose *r*_c_ to be equal to the cutoff distance for repulsive interactions between body beads, *σ*_BB_ = 138 nm.

To include a breakable bond with smooth energy and force properties at the breaking distance, we use a shifted Morse potential:

5 *U*(*r*) = *D*_e_[1 − exp(−*α*(*r* − *r*_eqm_))]^2^ − *D*_e_,

in which *D*_e_ is a “dissociation energy” representing the well depth, *α* is in units of inverse length and controls how steep the function is and *r*_eqm_ represents the equilibrium distance between two atoms. Unless otherwise specified, the equilibrium bond length parameter takes the value *r*_eqm_ = 50.0 nm, leading to a total polymer length of 300 nm, the steepness parameter takes the value *α* = 0.150 nm^−1^ and the dissociation energy takes the value *D*_e_ = 16.5 × 10^−21^ J. This equation has the properties that lim_*r*→∞_*U*(*r*) = 0 and 
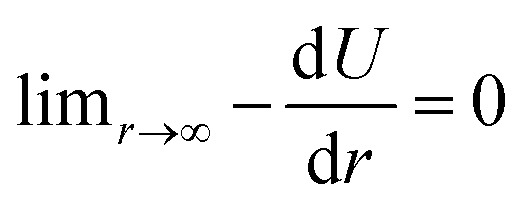
, meaning that the forces and energies in the system are unaffected if the breaking cutoff is set sufficiently large.

The cutoff distance can be no smaller than the excluded radius of a single body bead. The angular term at nodes is controlled by the balance between body bead repulsions and head bead attractions, so *σ*_BB_ is large with a value of 138 nm. Interactions between body beads in the same molecule are ignored, but when a bond breaks the beads are no longer considered part of the same molecule. If a bond breaks while two beads are within *σ*_BB_ of one another, they instantaneously feel an extremely large force and are pushed out of contact radius with one another. This destabilises the simulation, so it must be avoided by having a cutoff *r*_c_ > *σ*_BB_.

The bond breaking cutoff is independent of the potential parameters, so it may be that the bond energy is approximately zero for much shorter bonds than *r*_c_ which allows an “effectively broke” state before a bond is formally broken. However, the angular terms still apply to bonds which are “effectively broken”; if a group of 3 atoms are at 90.0° to one another and one bond breaks there will be a discontinuity in the energy as the angular potential disappears. For semi-stiff polymers this is not a major problem, because the timescale of a brittle fracture is short compared to the timescale of bending motion, and the relatively long persistence length of collagen IV molecules means that they can be treated as being approximately straight during this time.^30^ We also performed exploratory simulations with the 2D constraint removed, allowing the network to rumple. These simulations showed that the hexagonal network is broadly stable in 3D, adopting a width (measured by the standard deviation of *z* positions) of *σ*_*z*_ = 30.0 nm, approximately 10 .0% of the length of one molecule.

### 2.3 Linear stretching

To contextualise the mechanics of rupture in special cases, we must explore the landscape of potential rupturing modes in a simple scenario. The simple stretching simulation used here is to hexagonal network at a constant engineering strain rate 
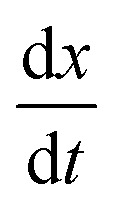
, expanding from a stretching ratio of *λ* = 1 to 2 over 10.0 μs, matching the range of stretching ratios used experimentally by Krag *et al.*^13^ We re-use much of the parameterisation from Bailey and Wilson as it allows studies of dynamics over large timescales (up to 100 μs).^12^ Idealised hexagonal networks were constructed with varying dissociation energies *D*_e_ and varying head group energies *ε* both in the range 1.00 × 10^−21^ J to 128 × 10^−21^ J at a temperature of 100 K (*k*_B_*T* ≈ 1.38 × 10^−21^ J). Each polymer represents a single tropocollagen molecule with a length of 300 nm. The parameterisation has been chosen to accelerate the damage to networks to fit within a computational timescale, which is considerably shorter than the physical timescales involved.

The simulations showed how the balance between bond breaking and head group breaking affected the mechanism of rupture. There were three final scenarios: “bead soup”, “polymer soup” and a damaged network. The first, “bead soup” occurred when *D*_e_ was small, meaning that bonds could break due to thermal oscillations. Alternately, when *ε* was small the polymers would drift apart and the bonds would stay rigid.

If bonds were weaker than head groups but stronger than thermal energy, a “mixed soup” arises where the body-head bonds preferentially break as they are under the most strain. The head beads then formed large clumps and the headless bodies floated free, remaining whole under little strain.

**Fig. 3:**
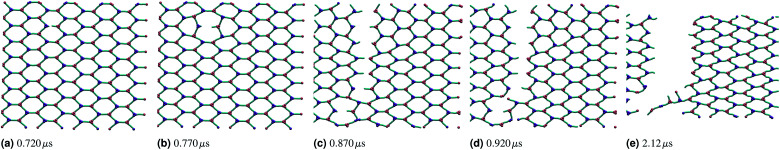
Snapshots shown at increasing simulation time and highlighting the progression of a rupture of a hexagonal polymer network stretched linearly (stretching increases across figures (a) through (e)). The rupture occurs initially randomly along one edge to relieve stress, and then opens up orthogonal to the stretching direction. When the rupture is the length of the periodic simulation cell, all stress is relieved and the remaining network is again purely hexagonal. The initial box size was 2.83 μm by 3.26 μm.

If the two energy scales were similar and larger than thermal energy, the networks showed interesting rupturing behaviour. This is shown in the panels of Fig. 3, each panel being successive snapshots of a rupturing network. In this network, one horizontal bond breaks initially. The energy of this break is dissipated into the network by two nearby hexagons relaxing to accommodate the decagon between them. This demonstrates the utility of a polymer molecular dynamics simulation to explore the rupturing mechanisms of soft materials, as energy can naturally be dissipated into both complex non-elastic network motion and motion of polymers. However, the top and bottom bonds of this decagon represent a new weak point in the network. As stretching continues, those bonds break and a tear rapidly propagates in a vertical line going through the original bond breaking site. A new rupture appears at the bottom left and joins the large tear; this gives the network enough flexibility to avoid tearing entirely, and a chain of bonds each with coordination number *k* = 2 holds the two network sections together before finally giving way.

**Fig. 4:**
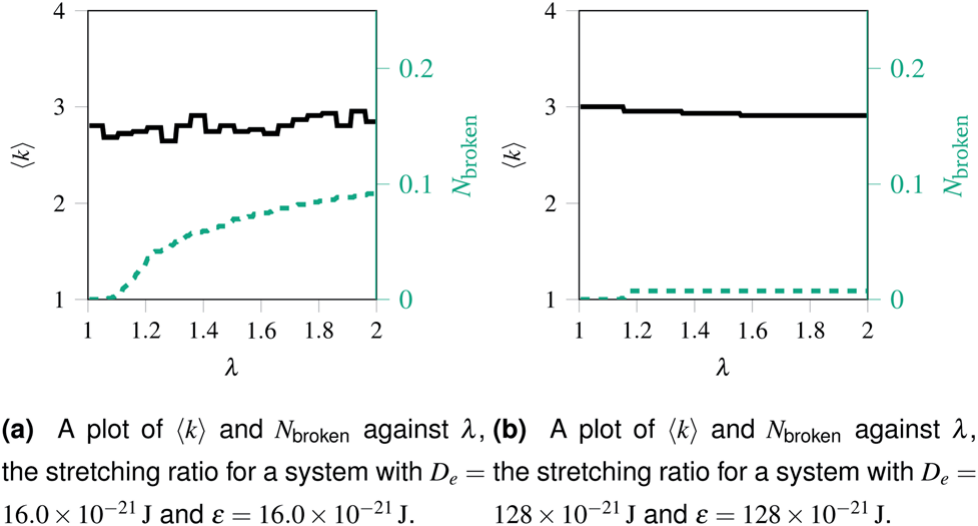
Damage at nodes and at bonds over time for two networks with different potential model parameters. The black solid line represents the mean node coordination number, <*k*>, which is 3 for an ordered hexagonal network and 1 for “loose” bonds. The red dashed lines represents *N*_broken_, the fraction of broken bonds, which is 0 for an unruptured network and 1 for a system with no remaining bonds.

Over each simulation we tracked a series of metrics: the average coordination number <*k*>, the fraction of broken bonds *N*_broken_, and the time until the first damage arose. Traditionally 2D networks are analysed using ring statistics, but those are unsuitable for the study of rupture. This is because rupture is a localised phenomenon, especially for a polymer network where separated polymers have minimal interactions. Those damage metrics are shown in Fig. 4b for the rupture shown in Fig. 3, and 4a for a network with weaker bonds. The average coordination number when rupture begins in Fig. 3b is 2.95, compared to 3.00 for an idealised hexagonal network leading to an average number of edges per polygon of 6.19 as opposed to 6. This is slightly lower than the average coordination number measured experimentally, which is in the range 3.20 to 3.40.^20^ The stretching ratio *λ* at which damage first occurs is approximate 1.15, matching well with modelling of disordered collagen networks performed by Burla *et al.*^15^

As the rupture continues the average node coordination is still very close to 3, and the average edges per polygon returns to 6 in Fig. 3e as the two halves of the network return to being two almost idealised hexagonal networks.

### 2.4 Sinusoidal stretches

The actual frequencies and amplitudes of the stretching that real networks undergo can be hard to match in simulation, as the natural motion of the eye takes place over seconds or milliseconds. Instead of applying a single maximum amplitude, we can simulate stretching networks at increasingly large amplitudes until they break. Such a simulation allows us to assess the initial breaking amplitude of a network and observe cumulative damage on an accelerated timescale. Changing the simulation cell naturally has the effect of pumping energy into the system, and it is partially this added kinetic energy that causes the damage. By changing the frequency of the simulation cell oscillations, it is possible to increase or decrease the amount of energy added to the network, and to change how much time the polymers in the network have to relax and accommodate their new arrangements.

The increasing sinusoidal simulations varied the box length *L*_*x*_ from its initial length Lx_0_ according to the equation

6 *L*_*x*_ = *L*_*x*_0__(1 + *A* sin(*ωt*))

with *A* the amplitude taking values in the range 0.100 to 0.500 and *ω* the frequency taking values in the range 0.100 μs^−1^ to 10.0 μs^−1^. These are faster than the oscillations observed by Liu *et al.* in the lens capsule after blunt trauma, which showed a roughly sinusoidal trend matching our *A* = 0.200 at a frequency of approximately 1.00 ms^−1^.^6^ The simulations were all run for a fixed time of 10.0 μs with the same potential model parameters of *ε* = 32.0 × 10^−21^ J and *D*_e_ = 32.0 × 10^−21^ J. This time and energy scale were chosen to encourage damage to the network over a short period of computational time.

**Fig. 5:**
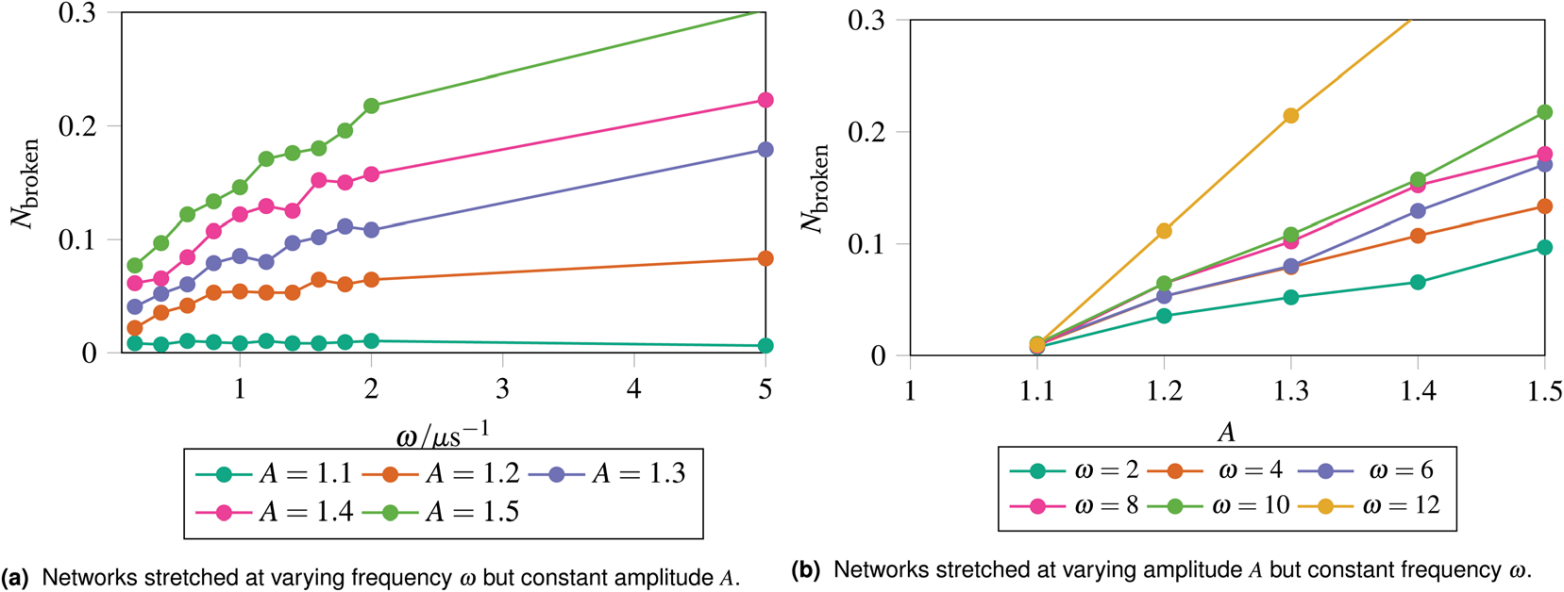
Recording the fraction of broken bonds at the end of a simulation as a function of stretching frequency *ω* and amplitude *A*. The fraction of broken bonds at the end of the simulation increases approximately linearly with both frequency and amplitude, as the most damage is introduced at the maxima and minima of a stretching cycle. The energy inserted into the network at the maxima and minima of stretching is linearly related to the frequency and amplitude.

The results are shown in Fig. 5, which shows the fraction of broken bonds as a function of (a) amplitude and (b) frequency of the applied oscillation. Fig. 5a shows the effect of varying frequency. At low amplitude (*A* = 1.1) the amount of damage is very low and approximately independent of *ω*. Put simply, if no damage occurs in an oscillation then the system can oscillate in perpetuity. The larger amplitude lines show that more damage has occurred by the end of the simulation; the damage is the least for the lowest frequency oscillations and the most for the highest frequency oscillations. The link between oscillation frequency and damage is roughly linear, although there are no samples between *ω* = 2 and *ω* = 5. In this range there is not an observable “tailing off” of the damage as *ω* increases, but instead damage increases monotonically with *ω*. The increased damage at higher frequencies is due to the increase in mechanical work being done to stretch the network, which can be used to break bonds within the network. We can also see the effect of increased mechanical work in Fig. 5b, where a larger stretching amplitude also leads to a larger amount of damage at each stretching frequency. The linear relation could be explained as the stretching forces are given by 
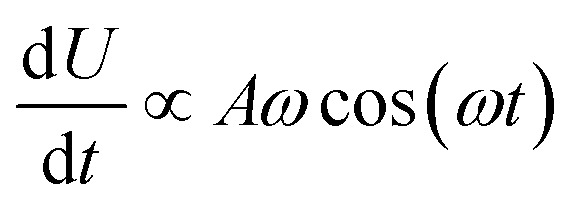
, which are maximised at ∝*Aω* at peaks and troughs of the stretching.

### 2.5 Edge removal defects

**Fig. 6:**
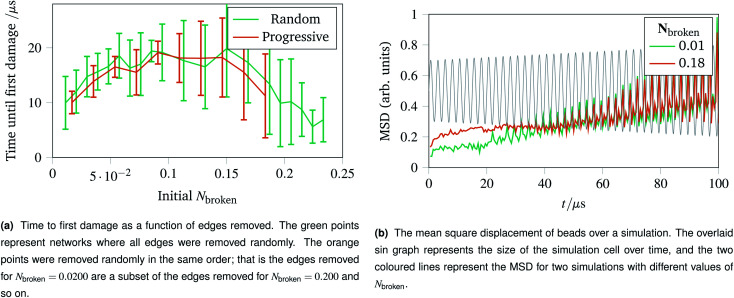
Time to first damage and the mean square displacement (MSD) of networks undergoing sinusoidal stretching with differing numbers of edges removed.

With the landscape of network damage explored in Sec. 2.3 and 2.4, we can now study how to provoke damage under controlled circumstances. As discussed in Sec. 2.1, one of the simplest forms of defect to introduce is a missing edge. The missing edge defects may provide a weak point in the network for larger ruptures to propagate from. Fig. 6a shows how long it takes for networks with *N*_broken_ bonds removed to experience their first irreparable damage when the simulation cell is stretched sinusoidally with increasing amplitude. Each data point represents an average over ten networks with a given fraction of edges removed. For the random edge removal, different edges were removed on each repeat. For the progressive edge removal, the same random seed was used such that all the networks had the same 0.0500 fraction of edges removed, and the edges removed for the 0.100 fraction simulations is a superset of the edges removed for the 0.0500 simulations. Each simulated network underwent 40 oscillations over 100 μs of linearly increasing amplitude up to a maximum of *A* = 1.5. The temperature was set to 100 K, and potential model parameters of *ε* = *D*_e_ = 16.0 × 10^−21^ J were used. Each set of simulation parameters was averaged over five instances.

This graph shows three clear regions:

• The flexible region from *N*_broken_ = 0 to 0.0750 where removing bonds allows more rearrangement, and thus slower damage.

• A noisy flattening off region from 0.100 to 0.150 where two effects counterbalance.

• A weakening region from 0.150 to 0.250 where the removed edges were structurally important.

The mean square displacement per bead as a function of time is shown in Fig. 6b, with the simulation cell stretching overlaid. This helps rationalise the difference in flexibility discussed above, because the network with more edges removed becomes coupled to the box gradually and less dramatically than the more rigid network with fewer edges removed.

**Fig. 7:**
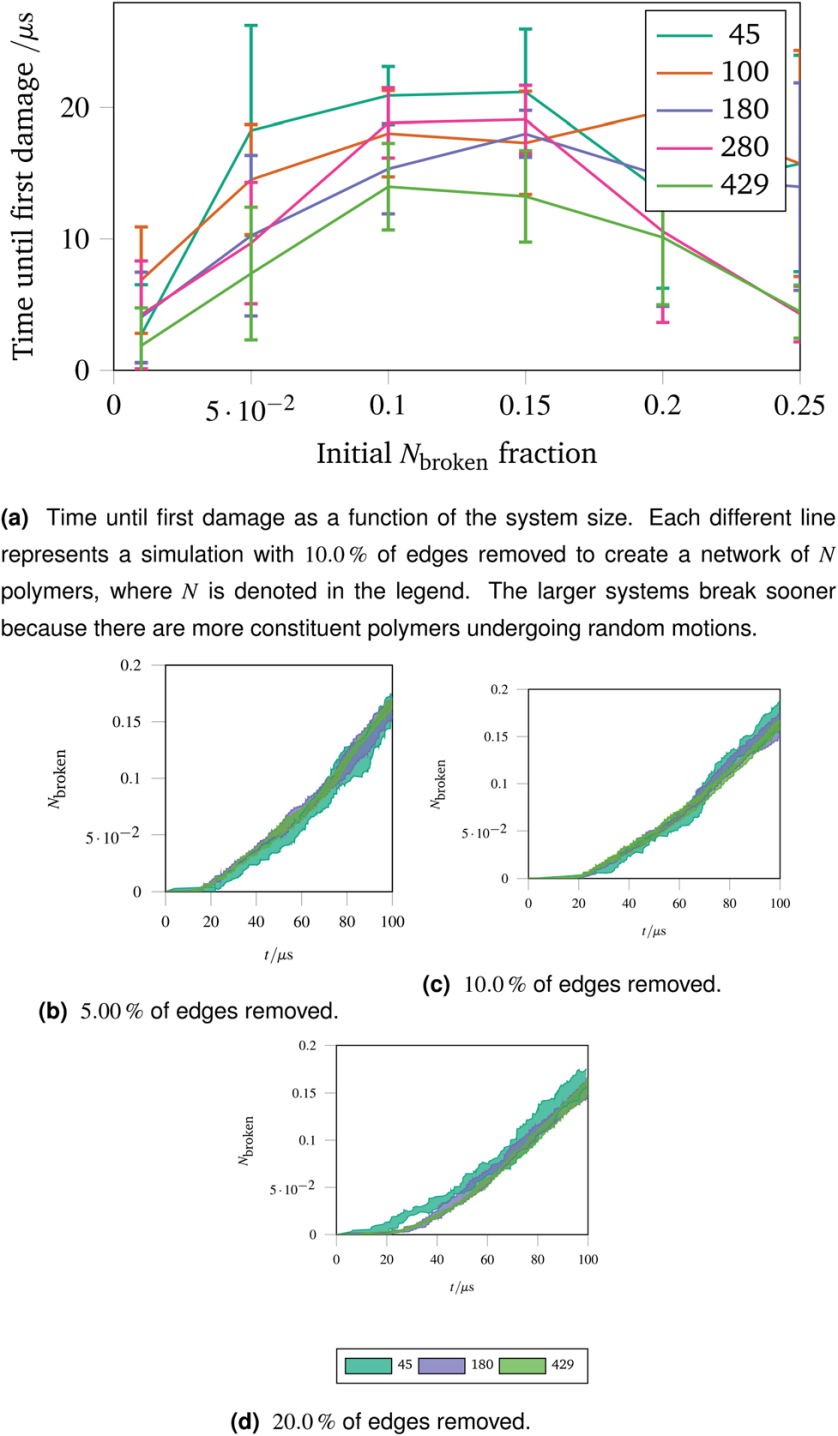
Damage trajectories for systems with the same fraction of edges removed but different system sizes. The shaded area in the bottom figures represents one standard deviation of the damage trajectory as described in the caption for Fig. 4a.

The importance of thermal damage can be seen by varying the size of the system while removing the same fraction of edges. A larger system will show damage sooner as there are more molecules at any given timestep with enough thermal energy to break. This is seen in Fig. 7a, where the larger systems show an earlier first damage (but with smaller error bars thanks to the central limit theorem). The smaller error bars also show the flexibility effect more clearly, with the curve observed in Fig. 6a being most clear for larger systems.

The shrinking of the uncertainty is clearer when a trajectory is analysed. Trajectories showing how the damage to a network evolves over time for networks with different fractions of edges removed are shown in Fig. 7b (with 5.00 % removed), Fig. 7c (with 10.0 % removed) and Fig. 7d (with 20.0 % removed). The time at which each network first develops damage (see Fig. 7a) is shown as the coloured lines leaving the *x* axis in the trajectories. Each sub-trajectory looks fundamentally similar but the shaded area (representing one standard deviation of the damage at each step) becomes smaller for larger systems as random events average out.

### 2.6 Combined defects

As discussed in Sec. 2.1, missing edges are not the only form of defect that is likely to occur in a polymer network. Previous work by Bailey *et al.* has shown that bond switching algorithms can generate networks that are similar to real biological networks.^22^ Bond switching methods can provide access to individual physically-informed defects in an ordered structure. To test the hypothesis that a small defect could prevent a tear (simulated by the edge removal defect) from propagating further, we combined single bond switching defects with edge removal defects studied in Sec. 2.5. We simulated networks with either one edge removed or one edge removed and a Stone–Wales defect added, undergoing sinusoidal stretching. This stretching was at a rate of 1.00 μs^−1^ over 10.0 μs for a total of 10.0 oscillations. The oscillations were up to a constant maximum amplitude of *A* = 1.1, and the simulation temperature was at 100 K. We varied the potential model parameters in the range *D*_e_ = 16.0 × 10^−21^ J to 64.0 × 10^−21^ J and *ε* = 16.0 × 10^−21^ J to 64.0 × 10^−21^ J, with *α* being kept constant at 0.150. Three different demonstration networks, before being transformed into polymers, are shown in Fig. 1.

**Fig. 8:**
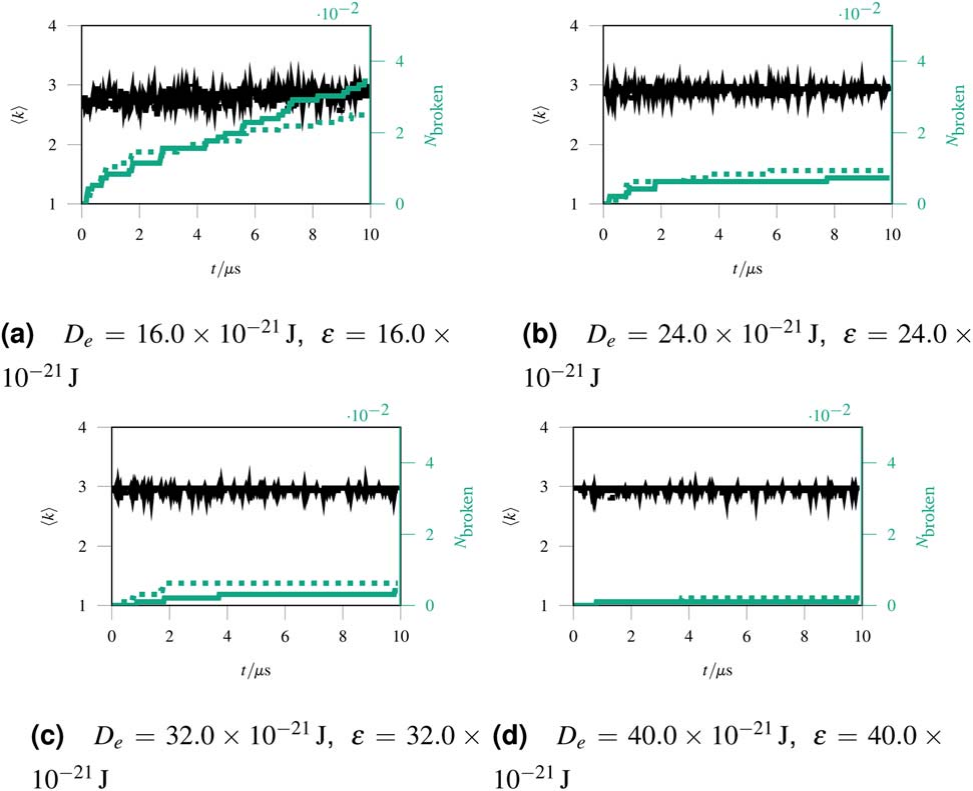
A comparison of networks with either just a removed edge (solid lines) or a removed edge and a Stone–Wales defect (dotted lines) stretched sinusoidally with different potential model parameters.

Results from these simulations are shown in Fig. 8, where no clear trend is visible. In some simulations such as Fig. 8a the double-defect network initially breaks more comprehensively than the edge-removed network, but the situation reverses at the end of the simulation with fewer broken bonds in the double-defect network. However, in Fig. 8b the opposite occurs. It does not appear that any additional flexibility afforded by the Stone–Wales defect prevents tears from propagating in this network. This is because the extra stress around the defect has a destabilising effect, which cancels out any stability from the flexibility around the defect. We anticipate that future work can further utilise the systematic approach to combining defects for a study of the rupturing properties of amorphous networks.

### 2.7 Central hole

**Fig. 9:**
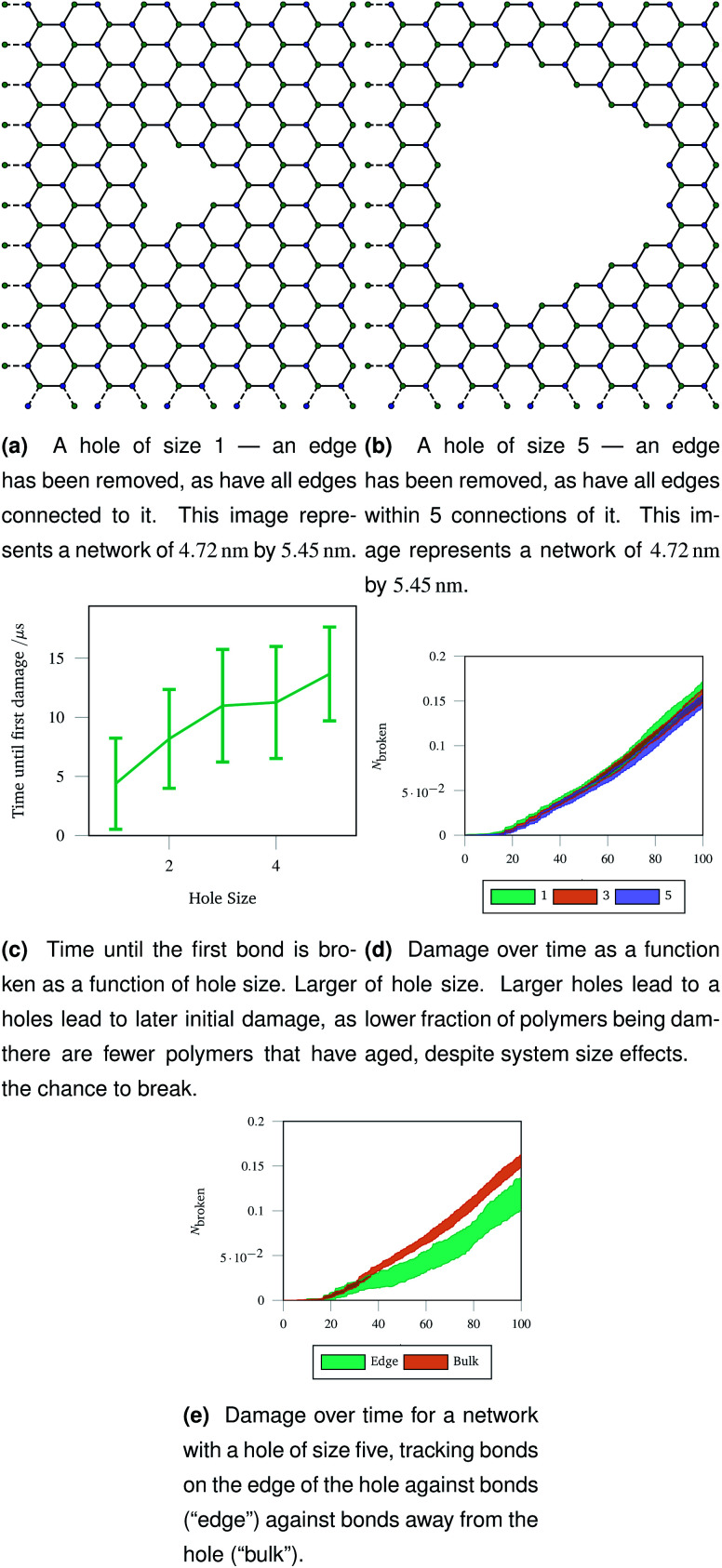
Graph representations of two networks with central holes of different sizes removed, and three metrics of damage resulting from stretching those networks. The damage metrics show the importance of flexibility around the central hole, as polymers near the edge break less, leading to less overall damage in a network with a larger hole removed.

An understanding of rupture mechanism during cataract surgery would be useful to identify potentially complicating factors before they happen. To mimic the rupture on a small scale, we created a hole in a hexagonal network by removing one node in the graph representation and all other nodes that were within a topological distance. The positions of nodes were then re-optimised and the graph representation was again converted to coarse grained polymers. Two example graph representations are shown in Fig. 9a and b with different sizes holes cut out of the hexagonal network. Each network underwent 40 oscillations over 100 μs, with a linearly increasing amplitude with a maximum of *A* = 1.5. The simulations were run at 100 K to avoid thermal effects breaking bonds. Each different hole size was simulated 20 times and averaged.

The networks were stretched as discussed in Sec. 2.4, and the same damage metrics were tracked. Networks with larger holes took longer for the first bond to break as seen in Fig. 9c, and suffered less damage both in absolute terms (number of molecules) and in relative terms (fraction of molecules) as time went on as shown in Fig. 9d. This is because the presence of the hole provides space for the rest of the network to deform into when stretched or compressed. Without this space to accommodate deformation, the network must rupture. The importance is space to accommodate deformation is demonstrated by the bonds on the edge of the hole being damaged less than bonds in the bulk of the network, away from the hole—this is shown in Fig. 9e for damage trajectories averaged over 20 simulations of a size 5 hole.

### 2.8 Thin strip defects

The surgical holes cut in collagen networks are extremely large compared to the length scale accessible to simulation. To match the exploration of rupturing mechanism around small holes discussed in Sec. 2.7, we simulated networks in the effective limit of an infinitely large hole. To mimic the infinitely large hole, we simulated a thin strip of idealised hexagonal network repeated periodically in the *y* dimension. We repeated the increasing oscillations simulation pattern used in Sec. 2.4. Each thin strip underwent 40 oscillations over 100 μs, with linearly increasing amplitude per oscillation up to a maximum of *A* = 1.5.

**Fig. 10:**
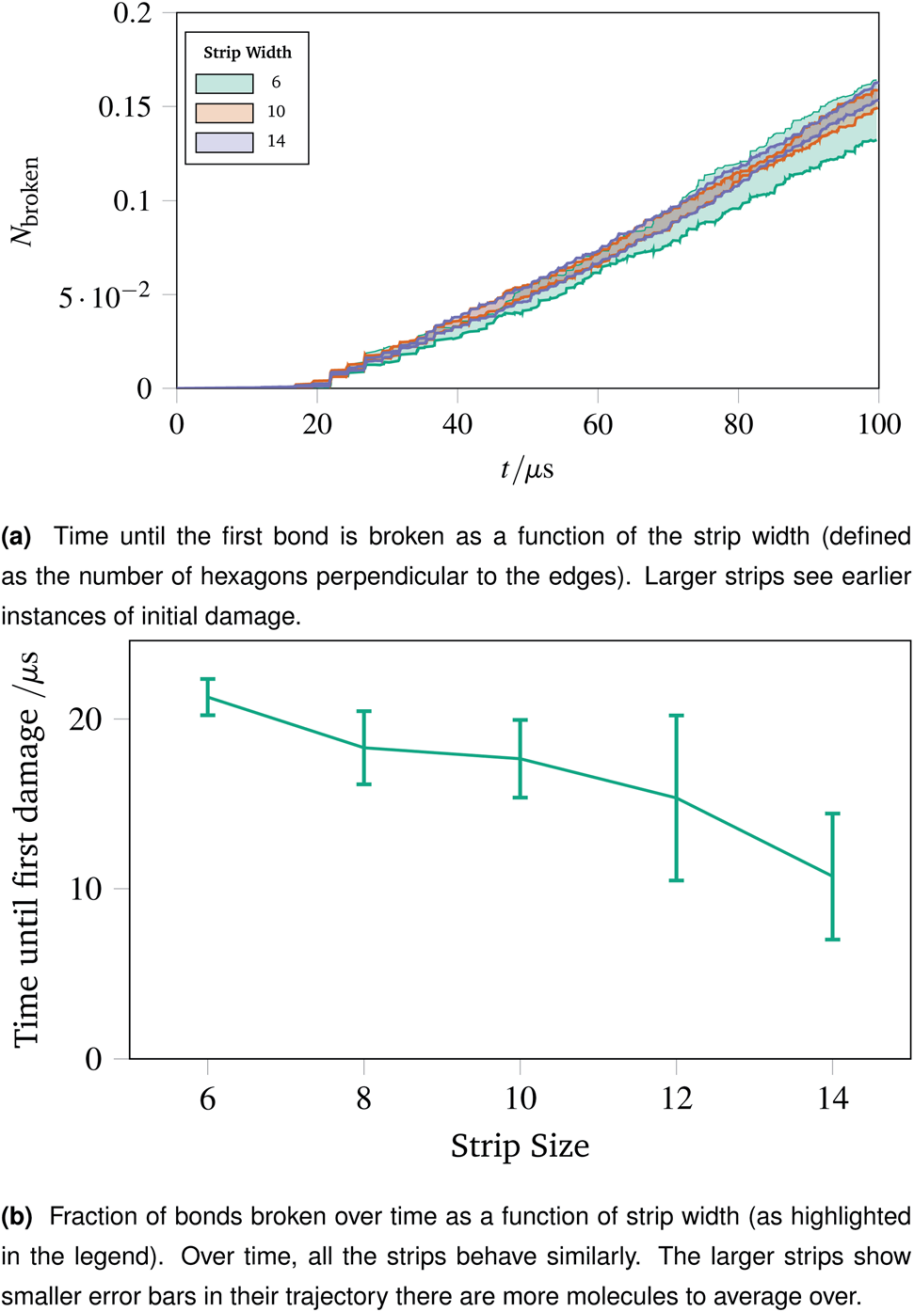
Time until first damage and the damage trajectory over time for different sizes of strip. The larger strips break sooner due to a larger number of constituent polymers, but the damage as a fraction of total bonds remains approximately constant.

Two metrics are shown in Fig. 10a and b. Fig. 10a shows how vulnerable the strips are to damage; the larger strips are damaged first. This is potentially because there are more molecules in the larger strips, each with their own thermal vibrations. With more molecules, the absolute rate of damage increases although the per-molecule rate stays similar. This effect is exacerbated as when the strips become larger, there are proportionally more bulk molecules compared to edge molecules.

The effect of the initial damage quickly evens out, as the damage over time in Fig. 10b shows. Here the trajectories show a similar fraction of molecules broken over time regardless of the size of the strip. This indicates reinforces the conclusion of Sec. 2.7, demonstrating that the room for a network to deform into is critical. The importance of space to deform into was seen previously with Fig. 9e, and the same effects are prevalent for thin strips, albeit less strongly, owing to the circular shape of the hole compared to a rectangular strip.

## 3 Conclusions

In conclusion, we have explored the mechanism of rupture in hexagonal polymer networks by subjecting those networks to a variety of different stretching scenarios. The models used are coarse-grained with the aim of mimicking the properties of collage IV networks, in particular, in the cornea.

We showed metrics that captured the mechanism of a simple rupture to a hexagonal network undergoing linear stretching, and demonstrated the utility of our metrics to quantify damage. More complex simulations explored sinusoidal stretching patterns, which mimic the actual strains the collagen network in the eye undergoes. We showed the link between stretching frequency or stretching amplitude and damage, accelerating the rate at which networks rupture to capture biologically relevant processes at achievable computational timescales.

We have simulated collagen networks with an increasingly complex array of combined defects to examine how this affects rupturing behaviour. Introducing edge removal defects showed that the flexibility in a network is critical in preventing damage, but there is a balancing factor where the networks weaken when too many edges are removed. We found that network flexibility is critical in preventing damage, although a network with too few links is weak and damages easily. Further, we introduced complicated defects such as the Stone–Wales defect representing a misplaced molecule, and combining that with edge removal defects. This showed that the flexibility around a defect would not prevent an existing rupture from propagating.

Finally, we mimicked the damage caused by surgery by studying the effects of networks with a central circular hole and thin strips of hexagonal network. These studies reinforced the importance of flexibility in the rupturing properties of a network.

All of these combined show the power of a systematic approach to introducing damage to a regular network, and the ability of a relatively simple coarse grained polymer model to capture large scale physical phenomena of chemical, biological and medical interest.

## Data availability

All code used to generate data in this paper is available on GitHub https://github.com/WilsonGroupOxford under the rings and WormLikeCurve repositories. This work made use of software packages including VMD, Networkx, Topotools, GNU Parallel and Numpy.^31–35^

## Author contributions

Matthew H. J. Bailey: data curation, investigation, software, visualization, writing – original draft; Mark Wilson: conceptualisation, project administration, supervision, writing – review & editing.

## Conflicts of interest

There are no conflicts to declare.

## Supplementary Material

## References

[cit1] Krag S., Andreassen T. T. (2003). Prog. Retinal Eye Res..

[cit2] Sueiras V. M., Moy V. T., Ziebarth N. M. (2015). Mol. Vision.

[cit3] Duane A. (1922). Am. J. Ophthalmol..

[cit4] Duncan G., Michael Wormstone I., Davies P. D. (1997). Br. J. Ophthalmol..

[cit5] David G., Pedrigi R. M., Humphrey J. D. (2017). Comput. Methods Biomech. Biomed. Eng..

[cit6] Liu X., Wang L., Du C., Li D., Fan Y. (2015). Comput. Methods Biomech. Biomed. Eng..

[cit7] Danysh B. P., Duncan M. K. (2009). Exp. Eye Res..

[cit8] Christie B. A. , McMaster B. M. and Blaker J. W., Accommodating Intraocular Lens, 1990

[cit9] Tsinopoulos I. T., Karras G. I., Haidich A. B., Tsaousis K. T. (2015). J. Cataract Refractive Surg..

[cit10] Ionides A. (2001). Br. J. Ophthalmol..

[cit11] Zhang Y., Debenedictis E. P., Keten S. (2019). Soft Matter.

[cit12] Bailey M. H. J., Wilson M. (2021). Comput. Struct. Biotechnol. J..

[cit13] Krag S., Olsen T., Andreassen T. T. (1997). Invest. Ophthalmol. Visual Sci..

[cit14] Jansen K. A., Licup A. J., Sharma A., Rens R., MacKintosh F. C., Koenderink G. H. (2018). Biophys. J..

[cit15] Burla F., Dussi S., Martinez-Torres C., Tauber J., van der Gucht J., Koenderink G. H. (2020). Proc. Natl. Acad. Sci. U. S. A..

[cit16] Burd H. J. (2009). Biomech. Model. Mechanobiol..

[cit17] Barnard K., Burgess S. A., Carter D. A., Woolley D. M. (1992). J. Struct. Biol..

[cit18] Yurchenco P. D., Ruben G. C. (1987). J. Cell Biol..

[cit19] Kalluri R. (2003). Nat. Rev. Cancer.

[cit20] Licup A. J., Münster S., Sharma A., Sheinman M., Jawerth L. M., Fabry B., Weitz D. A., MacKintosh F. C. (2015). Proc. Natl. Acad. Sci. U. S. A..

[cit21] Ormrod Morley D., Wilson M. (2018). J. Phys.: Condens. Matter.

[cit22] Bailey M. H. J., Ormrod Morley D., Wilson M. (2020). RSC Adv..

[cit23] Wooten F. O., Winer K., Weaire D. (1985). Phys. Rev. Lett..

[cit24] Virtanen P., Gommers R., Oliphant T. E., Haberland M., Reddy T., Cournapeau D., Burovski E., Peterson P., Weckesser W., Bright J., van der Walt S. J., Brett M., Wilson J., Millman K. J., Mayorov N., Nelson A. R. J., Jones E., Kern R., Larson E., Carey C. J., Polat İ., Feng Y., Moore E. W., VanderPlas J., Laxalde D., Perktold J., Cimrman R., Henriksen I., Quintero E. A., Harris C. R., Archibald A. M., Ribeiro A. H., Pedregosa F., van Mulbregt P., Vijaykumar A., Bardelli A. P., Rothberg A., Hilboll A., Kloeckner A., Scopatz A., Lee A., Rokem A., Woods C. N., Fulton C., Masson C., Häggström C., Fitzgerald C., Nicholson D. A., Hagen D. R., Pasechnik D. V., Olivetti E., Martin E., Wieser E., Silva F., Lenders F., Wilhelm F., Young G., Price G. A., Ingold G. L., Allen G. E., Lee G. R., Audren H., Probst I., Dietrich J. P., Silterra J., Webber J. T., Slavič J., Nothman J., Buchner J., Kulick J., Schönberger J. L., de Miranda Cardoso J. V., Reimer J., Harrington J., Rodríguez J. L. C., Nunez-Iglesias J., Kuczynski J., Tritz K., Thoma M., Newville M., Kümmerer M., Bolingbroke M., Tartre M., Pak M., Smith N. J., Nowaczyk N., Shebanov N., Pavlyk O., Brodtkorb P. A., Lee P., McGibbon R. T., Feldbauer R., Lewis S., Tygier S., Sievert S., Vigna S., Peterson S., More S., Pudlik T., Oshima T., Pingel T. J., Robitaille T. P., Spura T., Jones T. R., Cera T., Leslie T., Zito T., Krauss T., Upadhyay U., Halchenko Y. O., Vázquez-Baeza Y. (2020). Nat. Methods.

[cit25] Stone A. J., Wales D. J. (1986). Chem. Phys. Lett..

[cit26] Bamer F., Ebrahem F., Markert B. (2019). Comput. Mater. Sci..

[cit27] Mayo S. L., Olafson B. D., Goddard W. A. (1990). J. Phys. Chem..

[cit28] Plimpton S. (1995). J. Comput. Phys..

[cit29] Buehler M. J. (2006). Proc. Natl. Acad. Sci. U. S. A..

[cit30] Buehler M. J. (2006). J. Mater. Res..

[cit31] Humphrey W., Dlake A., Schulten K. (1996). J. Mol. Graphics.

[cit32] Hagberg A. A. , Schult D. A. and Swart P. J., 7th Python Sci. Conf. (SciPy 2008), 2008, pp. 11–15

[cit33] Kohlmeyer A. , Topotools, 2020, https://zenodo.org/record/3845031

[cit34] Tange O. , GNU Parallel, 2018, https://zenodo.org/record/1146014

[cit35] Harris C. R., Millman K. J., van der Walt S. J., Gommers R., Virtanen P., Cournapeau D., Wieser E., Taylor J., Berg S., Smith N. J., Kern R., Picus M., Hoyer S., van Kerkwijk M. H., Brett M., Haldane A., del Río J. F., Wiebe M., Peterson P., Gérard-Marchant P., Sheppard K., Reddy T., Weckesser W., Abbasi H., Gohlke C., Oliphant T. E. (2020). Nature.

